# Porcine pulmonary valve decellularization with NaOH-based vs detergent process: preliminary in vitro and in vivo assessments

**DOI:** 10.1186/s13019-018-0720-y

**Published:** 2018-04-25

**Authors:** Mathieu van Steenberghe, Thomas Schubert, Sébastien Gerelli, Caroline Bouzin, Yves Guiot, Daela Xhema, Xavier Bollen, Karim Abdelhamid, Pierre Gianello

**Affiliations:** 1 Pôle de Chirurgie Expérimentale et Transplantation (CHEX), Institut de Recherche Expérimentale et Clinique (IREC), Secteur des Sciences de la Sante, Université Catholique de Louvain, Avenue Hippocrate 55/B1.55.04, B-1200 Brussels, Belgium; 2 Service de chirurgie cardiaque et vasculaire, Clinique Cecil, avenue Louis Ruchonnet 53, 1003 Lausanne, Switzerland; 3 Service d’orthopédie et de traumatologie de l’appareil locomoteur, Cliniques universitaires Saint-Luc, Avenue Hippocrate 10, B-1200 Brussels, Belgium; 4 Unité de thérapie tissulaire et cellulaire de l’appareil locomoteur, Cliniques universitaires Saint Luc, Avenue Hippocrate 10, B-1200 Brussels, Belgium; 5 000 0004 0639 3167 Service de chirurgie cardiaque, Centre hospitalier Annecy-Genevois, site Annecy, 1 Avenue de l’Hopital, F-74370 Pringy, France; 6 Institut de Recherche Expérimentale et Clinique (IREC), IREC Imaging Platform (2IP), Université catholique de Louvain, Avenue Hippocrate 55/B1.55.20, B-1200 Brussels, Belgium; 7 Service d’anatomie pathologique, Cliniques universitaires Saint Luc, Avenue Hippocrate 10, B-1200 Brussels, Belgium; 8 Institute of Mechanics, Materials and Civil Engineering, Mechatronic, Electrical Energy, and Dynamic Systems (MEED), Secteur des Sciences et Technologies, Université Catholique de Louvain, Place du Levant 2/L5.04.02, B-1348 Louvain-la-Neuve, Belgium; 9 Service d’oncologie, Centre hospitalier universitaire vaudois, Rue du Bugnon 46, CH-1011 Lausanne, Vaud Switzerland

**Keywords:** Cardiovascular engineering, Heart valve, Xenograft, Decellularization, Biocompatibility, Remodeling

## Abstract

**Background:**

Glutaraldehyde fixed xenogeneic heart valve prosthesis are hindered by calcification and lack of growth potential. The aim of tissue decellularization is to remove tissue antigenicity, avoiding the use of glutaraldehyde and improve valve integration with low inflammation and host cell recolonization. In this preliminary study, we investigated the efficacy of a NaOH-based process for decellularization and biocompatibility improvement of porcine pulmonary heart valves in comparison to a detergent-based process (SDS-SDC0, 5%).

**Methods:**

Native cryopreserved porcine pulmonary heart valves were treated with detergent and NaOH-based processes.

Decellularization was assessed by Hematoxylin and eosin/DAPI/alpha-gal/SLA-I staining and DNA quantification of native and processed leaflets, walls and muscles.

Elongation stress test investigated mechanical integrity of leaflets and walls (*n* = 3 tests/valve component) of valves in the native and treated groups (*n* = 4/group).

Biochemical integrity (collagen/elastin/glycosaminoglycans content) of leaflet-wall and muscle of the valves (n = 4/group) was assessed and compared between groups with trichrome staining (Sirius Red/Miller/Alcian blue).

Secondly, a preliminary in vivo study assessed biocompatibility (CD3 and CD68 immunostaining) and remodeling (Hematoxylin and eosin/CD31 and ASMA immunofluorescent staining) of NaOH processed valves implanted in orthotopic position in young Landrace pigs, at 1 (*n* = 1) and 3 months (*n* = 2).

**Results:**

Decellularization was better achieved with the NaOH-based process (92% vs 69% DNA reduction in the wall). Both treatments did not significantly alter mechanical properties. The detergent-based process induced a significant loss of glycosaminoglycans (*p* < 0,05).

In vivo, explanted valves exhibited normal morphology without any sign of graft dilatation, degeneration or rejection. Low inflammation was noticed at one and three months follow-up (1,8 +/− 3,03 and 0,9836 +/− 1,3605 CD3 cells/0,12 mm^2^ in the leaflets). In one animal, at three months we documented minimal calcification in the area of sinus leaflet and in one, microthrombi formation on the leaflet surface at 1 month. The endoluminal side of the valves showed partial reendothelialization.

**Conclusions:**

NaOH-based process offers better porcine pulmonary valve decellularization than the detergent process. In vivo, the NaOH processed valves showed low inflammatory response at 3 months and partial recellularization. Regarding additional property of securing, this treatment should be considered for the new generation of heart valves prosthesis.

**Graphical abstract:**

Graphical abstract of the study
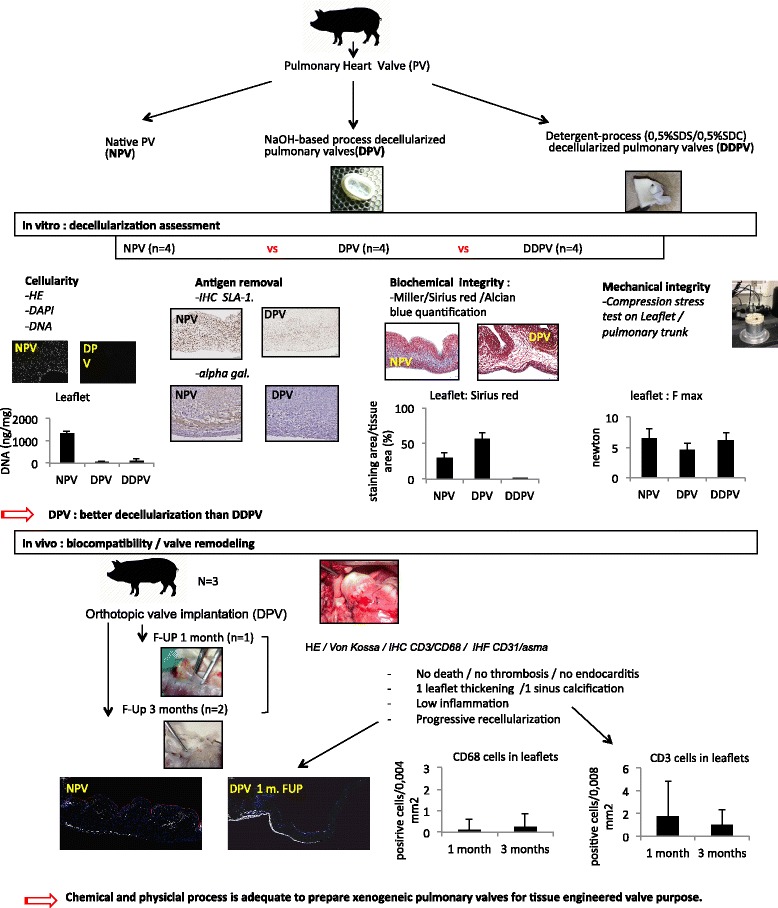

## Background

Currently, heart valve prostheses are hindered by several limitations. Mechanical valves have excellent long-term durability but require lifelong anticoagulation therapy due to thromboembolic risks. Bioprostheses do not require anticoagulation but show reduced durability and are more prone to degeneration, particularly in younger patients. This phenomenon has been related to a more reactive immunity, higher calcium and phosphate metabolism and physical activity that play a role in prosthesis calcifications [1]. Bioprosthetic valves are usually treated with glutaraldehyde to prevent immune rejection of the xenogeneic scaffold. But it has been early shown that glutaraldehyde modifies mechanical properties of the native tissue, is cytotoxic, does not remove tissue phospholipids, induces a release of cell debris, and does not completely suppress the immune reaction against the graft. These events lead to chronic inflammation and calcifications [1–5].

Finally, cryopreserved homografts with ideal hemodynamic performance but limited availability also show limited durability because of residual tissue immunogenicity [6–9]. Mechanical and bioprosthetic valves share another disadvantage: they cannot grow and remodel, therefore resulting in subsequent revision surgeries in pediatric patients [10].

Decellularization of biological valves is an alternative. This concept involves removing all cellular components that are supposedly immunogenic and may lead to calcifications while minimizing any adverse effect on the composition, biological activity, and mechanical integrity of the matrix. The resulting extracellular matrix (ECM) can be recellularized by the host and functionally and structurally integrated into the body with growth potential [11].

As of today, new products are already commercially available but short term results with some of those implants, essentially those of xenogeneic source, did not demonstrate convincing results in the pediatric population [12, 13], while others, essentially decellularized homografts from allogeneic source seemed to yield better midterm term results [14, 15].

Despite these progresses, no gold standard decellularization process exists. Various decellularization protocols are proposed in the literature and most popular are those using detergents [16, 17].

We previously demonstrated enhanced biocompatibility and vascular remodeling of allogeneic and xenogeneic pericardium with a treatment based on NaOH decellularization in comparison to the glutaraldehyde fixation and detergent process [18, 19].

This NaOH-based process has the particularity of being inactivator for conventional (virus/bacteria) and non-conventional (prion) pathogens and therefore improves the security of those grafts [20–22].

We investigated this treatment as a decellularization process to improve biocompatibility and remodeling of xenogeneic pulmonary heart valves for tissue engineering applications.

In vitro experiments assessed decellularization, antigen removal, mechanical and biochemical integrity of porcine pulmonary heart valves treated with the NaOH-based process (decellularized porcine pulmonary heart valves with NaOH based-process: DPV) as well as porcine pulmonary heart valves treated with a conventional detergent process (decellularized porcine pulmonary heart valves with detergent-based process: DDPV) and native porcine pulmonary heart valves (NPV). Moreover, an in vivo preliminary study was conducted to assess biocompatibility (inflammation and calcifications) and remodeling (endothelialization and recellularization) at 1 and 3 months follow-up of DPV valve in a growing allogeneic/porcine model.

## Methods

### Sources of matrices

For in vitro study, porcine Landrace hearts were procured from a local slaughterhouse (Eurovlees, Zele, Belgium).

For in vivo study, porcine hearts were procured at our laboratory, from Landrace pigs weighing 20 kg, used for abdominal surgery course/demonstration. Animals were euthanized, and hearts harvested respecting the standards of animal care. The pulmonary valves were then harvested, rinsed with sterile ringer solution and frozen at − 80 °C.

### Matrix preparation

Before processing, valves were thawed and washed in sterile ringer solution.

Two treatment protocols were conducted. The detergent-based process was based on a conventional detergent protocol previously published. Briefly, porcine pulmonary valves were incubated for 48 h in an aqueous solution containing 0.5% sodium deoxycholate (SDC) and 0.5% sodium dodecyl sulfate (SDS) under continuous agitation followed by a 72 h rinsing step in a continuous flow of demineralized water [23, 24].

As previously described, the NaOH-based process consists of a succession of baths of acetone, ethanol, NaOH and Hydrogen Peroxyde. This chemical treatment ensures defatting, prions/viruses and bacterial inactivation [18].

**Fig. 1:**
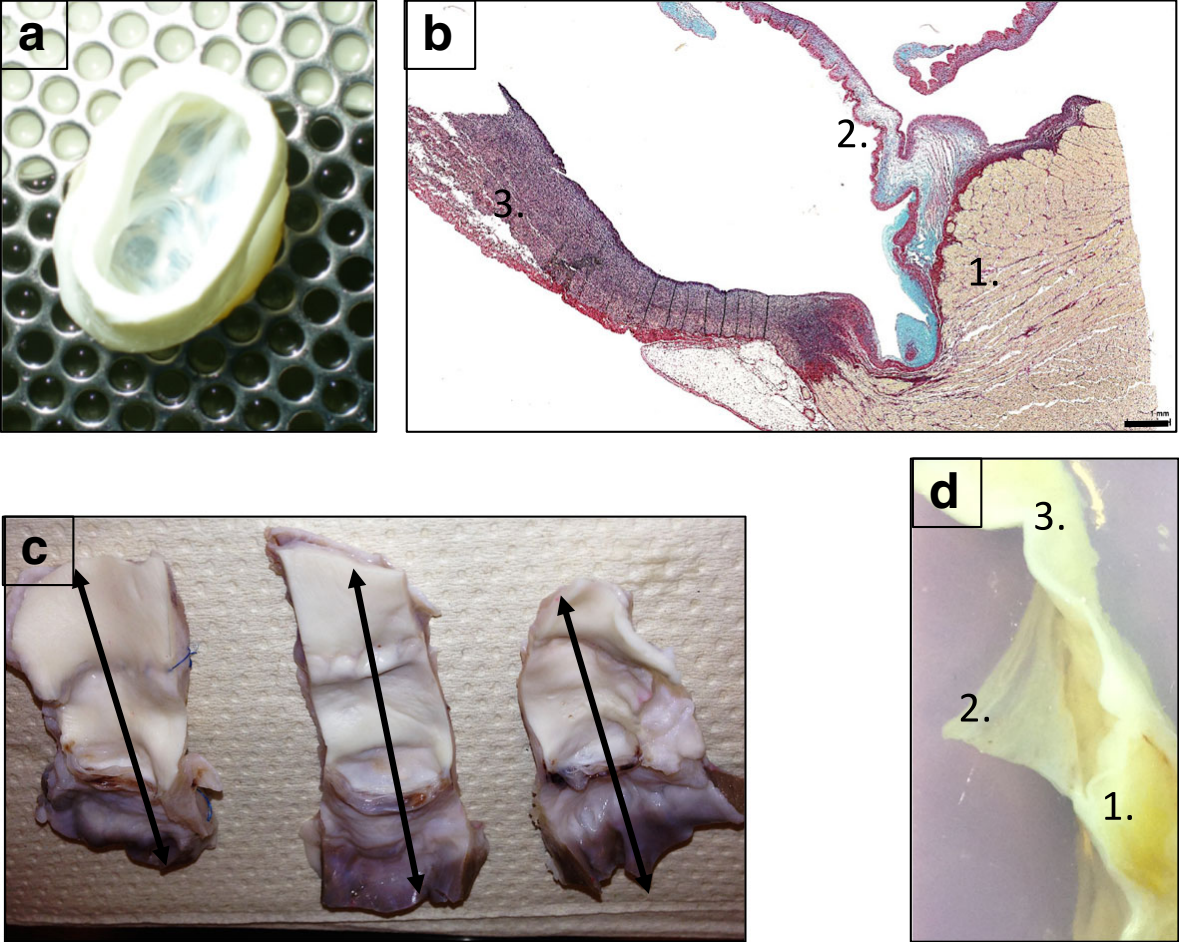
Valve characterization: methods. **a**: macrophotograph of a DPV. **b**: microphotograph of NPV after trichrome staining illustrating histological assessment of the valves in 3 zones:1: the muscle/2: the leaflet/3: the wall. Scal barr: 1 mm. For histological evaluation, the explanted valves were sectioned in six segments (**c**) to analyze the three zones (**d**)

Finally, the valves (Fig. 1a) were frozen at − 80 °C without solution and individually packed.

### In vitro characterization

Valves were individually characterized for leaflet, pulmonary trunk (wall) and muscle structure.

For histological assessment, the valves were cut longitudinally to explore wall, leaflet and muscle on the same slide (Fig. 1b). The slices were immediately fixed overnight in 4% formaldehyde and embedded in paraffin. Serial sections (thickness of 5 μm) were mounted on glass and dried for 12 h at 37 °C.

#### Hematoxylin and eosin and DAPI staining

Decellularization was evaluated first by Hematoxylin and eosin (HE) and 40.6-diamidino-2-phenylindole (DAPI; 1 μg/ml/Abbot Molecular Inc-USA) staining. These two methods reveal the nuclei. Sections from 4 different valves per condition (NPV, DPV, DDPV) were stained and slides were then digitalized using a SCN400 slide scanner (Leica Biosystems, Wetzlar, Germany) at 20× magnification for HE. For DAPI assessment, samples were photographed at high definition under structured illumination using a Zeiss AxioImager-Apotome system.

#### Antigen removal

The presence of residual antigens such as alphagalactosyltransferase antigen (alpha-gal) and swine leucocyte antigen class 1 (SLA-1: the major histocompatibility complex class1 region of pigs) were investigated by immunohistochemistry. Four valves per condition (NPV, DPV, DDPV) were analyzed. After deparaffinization, endogenous peroxidases were inhibited during a 20-min methanol bath with 3% hydrogen peroxide. Primary antibodies (mouse anti-SLA class Ic (1:200; 16.7. E4.2: IgM) [25]); mouse anti-alpha galactose, M86, Enzo Life Sciences®) were revealed with the corresponding Envision HRP-coupled antibodies (Dako) and Diaminobenzidine staining. After counterstaining with hematoxylin, slides were dehydrated and mounted. Stained slices were then digitalized using a SCN400 slide scanner (Leica Biosystems, Wetzlar, Germany) at 20× magnification.

#### DNA quantification

DNA was extracted with DNeasy® Blood & Tissue Kit (QIAamp DNA Mini Kit, QIAGEN, Hilden, Germany). Three valves per condition were used and one sample per valve region was processed. Extracted DNA was quantified by Quant-it Picrogreen DNA assay kit (Invitrogen, CA, USA), according to manufacturers’ protocol. Fluorescence was read at 480 nm and 520 nm. Final DNA concentration was expressed in ng/mg dry weight.

#### Mechanical properties

Uniaxial mechanical resistance tests were performed on a minimum of four valves per condition on the three leaflets and three samples of pulmonary wall per valve. Samples of 15 mm on 15 mm for wall and the whole leaflets were placed between two plastic structure with a central hole of 8,5 mm diameter where a probe comes in contact and applies pressure on the tissue. Mechanical testing was performed, using an Instron traction system with Instron bluehill software (Model 5600, Instron, Canton, MA) with a load-to-failure test set at an elongation rate of 3 mm.min^− 1^. The load to elongation behavior of the matrices and failure modes were recorded. The structural properties of the matrices were represented by stiffness (Nm.m^− 1^) and ultimate load (N). Stiffness (k) was calculated as k = ΔF/ΔL where, *F* is the force applied on the body and L is the displacement produced by the force along the same degree of freedom. These parameters were compared between native and treated tissues. Tests were not conducted on muscle.

#### Biochemical integrity

Longitudinal slices from 4 different valves per condition (NPV, DPV, DDPV) were analyzed. Five micrometer sections were stained using a combined Miller, alcian blue and sirius red trichrome, as described by Sarathchandra P. [26]. The Miller stains elastin in dark blue, the alcian blue colors the glycosaminoglycans (GAGs) in Cyan and the sirius red stains collagen in red.

Quantification was performed individually on leaflet, pulmonary wall and pulmonary trunk using Tissue IA software (Leica Biosystems, Dublin, Ireland). Pixels corresponding to the Miller, Alcian blue and Sirius red staining were selected separately to create three color profiles. Total tissue area was defined by setting an intensity threshold (grey value). Results were expressed as a percentage of stained area and calculated as (stained area/tissue area) × 100.

### In vivo study

#### Surgical procedure

Animals were housed according to the guidelines of the French Ministry of Agriculture and Animal Care. All procedures were approved by the local Ethics Committee for Animal Care of the Ecole de Chirurgie - Université de Lorraine, Nancy (D57–547-5).

Three female Landrace pigs weighting 40 kg were kept unfed for 24 h before the operation. A premedication of ketamine (1000 mg) was administered by IM. The animals were then intubated and kept under general anesthesia throughout the operation. A physiological follow-up (oximetry, pulse, and heart rate) was conducted throughout the entire operating procedure. After longitudinal sternotomy, the heart was exposed. Systemic heparinization was achieved with an activated coagulation time of 400 s. Pediatric cardiopulmonary by-pass was then placed and turned on. Then, the native pulmonary artery root was harvested and replaced with DPV with two 5.0 prolene running sutures. After implantation, the pigs were weaned off bypass. The cannulas were removed and the sternum was closed. The pigs received low molecular weight heparin prophylaxis (40 mg/day) for 5 days. The animals did not receive antibiotics. One pig was euthanized at day 30 and 2 pigs were euthanized at day 90. The valves were then removed and cut in order to obtain three parts relating to posterior, right and left leaflets and the corresponding sinus, pulmonary wall and muscular base. Finally, these portions were divided in two parts to obtain six segments (Fig. 1c/d). Tissues were fixed overnight in 4% formaldehyde and embedded in paraffin.

#### Histological evaluation

##### Coloration and staining

Hematoxylin and eosin, Masson’s trichrome and von Kossa stainings assessed remodeling/cell infiltration, structure and calcifications respectively.

Immunohistochemistry for CD3 and CD68 were performed using a Ventana Benchmark XT machine (Roche®, USA) to assess inflammatory reaction. The CD 68 is particularly useful as a marker for the various cells of the macrophage lineage, including monocytes, histiocytes, giant cells, Kupffer cells. CD3 is highly specific of all stages of T-cell development [18]. Slides were digitalized at 20× magnification with a SCN400 slide scanner (Leica, Wetzlar, Germany) and visualized on the Digital Image Hub (Leica Biosystems, Dublin, Ireland).

For immunofluorescent co-staining, 5 μm sections were subjected to endogenous peroxidases inhibition for 20 min and then to specific antigen binding sites for 1 h (PBS with 5% BSA and 0.05% Triton). Rabbit anti-CD31 (polyclonal, Abcam, # ab28364, 1/100 dilution for rat, 1/50 dilution for pig) and Mouse anti-ASMA (clone 1A4, Abcam #ab7817, 1/100 dilution, for pig), primary antibodies were incubated overnight at 4 °C in PBS containing 1% BSA and 0.05% Triton X-100. This was followed by an incubation with AlexaFluor 568 anti-rabbit and AlexaFluor647 anti-mouse (Invitrogen) secondary antibodies, incubated at a 1/1000 dilution for 1 h at room temperature. Nuclei were stained with DAPI and labeled sections.

Stained sections were digitized using a Pannoramic P250 FlashIII slide scanner (3DHistech) at 20× magnification and visualized using CaseViewer.

##### Histomorphometry

A minimum of five regions of interest [ROI] in the three parts (wall/leaflet/muscle) of the six segments of the explanted DPV were analyzed at × 20 magnification with a grid representing a surface of 0.12 mm square. CD3 and CD68 immunohistochemical staining’s were assessed by point counting as previously described [18].

### Statistical analysis

One-sample Kolmogorov–Smirnov tests and QQ-plots were used to ensure the normal distribution of values. Results were expressed as means ± SD or in ratios. The statistical significance of differences between experimental groups was tested by Student-T or one-way analysis of variance with a Bonferroni’s post hoc test. The statistical tests were carried out with PASW 18. Differences were considered to be significant at *p* < 0.05.

## Results

### In vitro characterization

#### Decellularization and antigen removal

Staining for HE, DAPI and alpha-gal and SLA-I was positive for controls in the three parts of the valve.

**Fig. 2:**
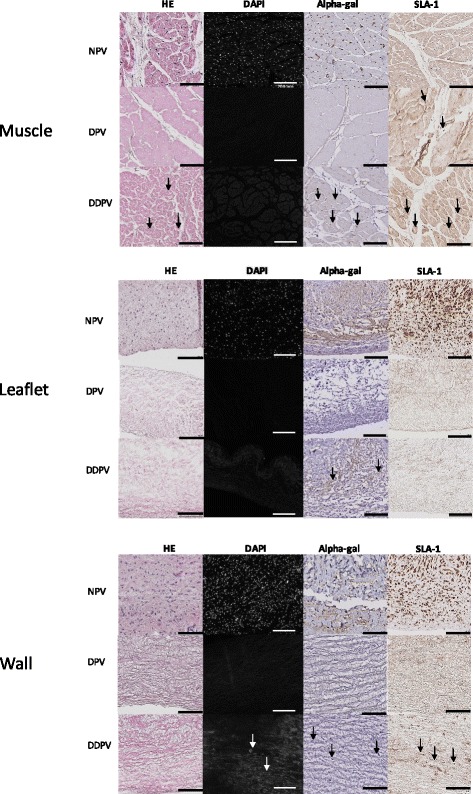
Histological valves characterization: Hematoxylin and eosin/DAPI/Alpha-gal and SLA-1 staining. Representative histology microphotograph of muscle (first table)/leaflet (second table) and wall (third table) of NPV (first line), DPV (second line) and DDPV (third line) after Hematoxylin and eosin (HE, first column), DAPI (second column) Alpha-gal (third column) and SLA-1 (fourth column) staining. (Black scale bar: 100 μm and white scale bar: 200 μm). The black arrows show positive staining. The staining for NPV (both muscle/leaflet and wall) was positive in all conditions (HE/DAPI/Alpha-gal and SLA-1) and was clear

Decellularization and antigen removal were more complete for DPV than for DDPV as well for the muscle, wall or leaflet of treated valves. Positive staining for alpha-gal and SLA-I were still detected for DDPV in the three parts. Cells were also evidenced with hematoxylin and eosin for DDPV muscle and wall (Fig. 2/Table 1).

**Table 1:** Assessment of HE/DAPI/Alpha-gal and SLA-1 stainings for muscle/leaflet/wall of control (NPV, *n* = 4), DPV(n = 4) and DDPV(n = 4)

		H.E.	DAPI	Alpha-gal	SLA-1
Muscle	NPV	+++	+++	+++	+++
	DPV	–	–	–	+
	DDPV	+	–	+	++
Leaflet	NPV	+++	+++	+++	+++
	DPV	–	–	–	–
	DDPV	–	–	+	–
Wall	NPV	+++	+++	+++	+++
	DPV	–	–	–	–
	DDPV	+	+	+	++

Semi-quantitative numerical scale: –: no staining; +: staining traces; ++: moderate staining and +++: intense staining

#### Mechanical integrity

**Fig. 3:**
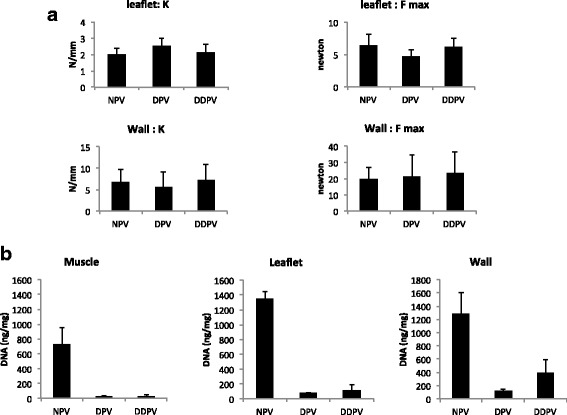
Valves characteristics: Mechanical properties and DNA content. **a**: Stiffness (K: N/mm) and maximal load before rupture (F max: Newton) of leaflet and wall of NPV, DDPV and DPV. No significant differences were observed between groups. **b**: DNA content (ng/mg) in muscle, leaflet and wall of NPV, DDPV and DPV

No differences between NPV, DPV and DDPV regarding elasticity and maximal load of leaflets or pulmonary wall were detected (Fig. 3a).

#### DNA

Better DNA reduction was achieved with NaOH-based process in comparison with detergent process. The DPV leaflets showed 95% DNA reduction to NPV while DDPV showed 92% DNA reduction. In the wall of DDPV, DNA content was reduced to 69% and in the DPV, the content was reduced to 92%. In the muscle, DNA reduction was quite similar for both treatments: 96% for DDPV vs 97% for DPV (Fig. 3b/Table 2).

**Table 2:** DNA content (ng/mg) in NPV, DDPV, DPV and DNA reduction in DDPV and DPV

	Native (n = 3)	DDPV (n = 3)		DPV (n = 3)	
	Total DNA (ng/mg)	Total DNA (ng/mg)	% reduction	Total DNA (ng/mg)	% reduction
Muscle	730,58+/−223,20	31,02+/− 20,07	96%	23,35+/− 18,92	97%
Leaflet	1345,65+/−100,64	110,3231+/−85,27	92%	77,16+/−7,94	95%
Wall	1278,85+/− 332,05	396,60+/−196,30	69%	122,34+/−23,52	92%

#### Biochemical integrity

##### Muscle

**Fig. 4:**
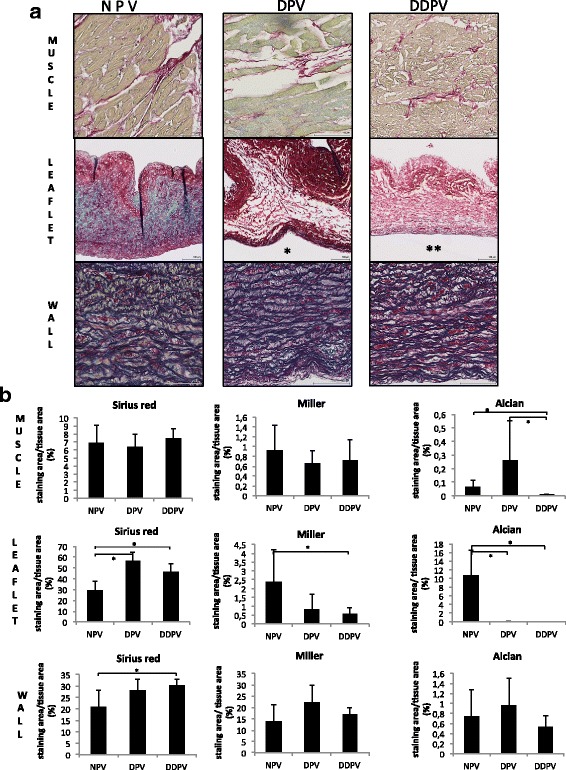
Biochemical valves characterization. **a**: Table of representative histologic microphotographs of the three parts (muscle/leaflet and wall) of NPV (first column), DPV (second column) and DDPV (third column) after trichrome staining. Scale bar: 100 μm. Note reduction of blue staining (GAGs) in treated leaflets (*: DPV leaflet and **: DDPV leaflet) while coloration of wall in the different conditions are quite similar. **b**: Quantification of staining area for Sirius red (collagen), Miller (elastin) and Alcian blue (GAGs) /analyzed tissue area in muscle (first line) /leaflet (second line) and wall (third line) of NPV, DPV and DDPV after trichrome staining. *: *p* < 0.05

Collagen and elastin staining were maintained in DPV and DDPV in comparison to NPV. However, significant reduction of GAGs staining in DDPV muscle in comparison to NPV occurred (*p* = 0.021) while there was no difference between DPV and NPV (Fig. 4).

##### Leaflet

Histological examination after trichrome staining revealed evidence of GAGs staining reduction after both treatments. Software analysis showed GAGs staining was significantly reduced for both DPV and DDPV to NPV with *p* < 0.05 while elastin staining was significantly reduced for DDPV to NPV (p = 0.021).

Collagen staining was not reduced after both treatments (Fig. 4).

##### Wall

No significant reduction of staining was noticed for DPV and DDPV in comparison to NPV (Fig. 4).

### In vivo study

No deaths occurred. The three pigs showed regular growth (to reach 60 kg at 1 month and 120 kg at 3 months).

### Macroscopic evaluation

The explanted valves exhibited no signs of graft dilatation, degeneration or rejection. The luminal surface of the arterial wall was similar to the adjacent host artery.

**Fig. 5:**
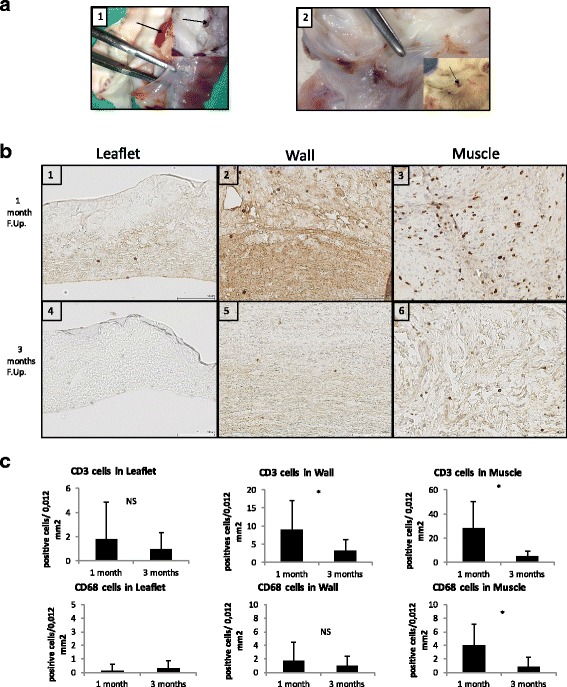
DPV in vivo results: macroscopic assessment and CD3/CD68 infiltration. **a**: Macroscopic views of explanted valves at one month (1) and three months (2) follow-up. The forceps indicate translucent and flexible leaflets. The black arrow in 1 shows thrombi formation and in 2, a micro calcification in the posterior sinus. **b**: Representative histological findings of CD3 staining of DPV at one month (first line) and three months (second line) of follow-up in leaflet (1;4), wall (2;5) and muscle (3;6). Note staining was the highest in muscle at one month (3) and reduction of staining at three months in comparison to one month (1- > 4 for leaflets/2- > 5 for wall and 3- > 6 in muscle). Scale bar: 100 μm. **c**: Results of histomorphometry for CD3 (first line) and CD68 (second line) staining in the three portions of DPV at one month and three months showing a decrease of inflammatory reaction at three months in all portions of the valve and significantly (*: p < 0,05) in wall and muscle portions for CD3 infiltration and in wall portion for CD68 infiltration. Low inflammation in the leaflet, the wall and muscle portions was found at 3 month: for CD3 infiltration: respectively 0,98 +/− 1,36; 3,1+/− 3,28 and 5,08 +/− 4,35 cells/0,12 mm^2.^ and for CD68 infiltration: respectively CD68: 0,32 +/− 0,69; 0,93 +/− 1,44 and 0,87 +/− 1,34 cells/0,12mm^2^)

At 1 month, the DPV exhibited two translucent, flexible and mobile leaflets while one small thrombus was detected in the third one (Fig. 5a1).

At 3 months, all leaflets of the two DPVs were translucent, flexible and mobile. We noticed one calcification in one posterior leaflet sinus (Fig. 5a2).

#### Histological evaluation

##### Inflammation

CD3 infiltration was essentially located in the muscle at 1 month. The CD3 count was significantly the lowest in the leaflets (*p* < 0,005). At 3 months, the CD3 infiltration showed the same repartition than at 1 month with the highest count in the muscle with p < 0,05. At 3 months in comparison to 1 month, infiltration was significantly reduced in the wall and in the muscle with p < 0,005 while leaflet infiltration was still low (0,98+/− 1,36 cells/0,12 mm^2^) (Fig. 5b/c).

At 1 month CD68 infiltration was low in all parts of the valves with the highest count in the muscle part with p < 0,05. The lowest count was in the leaflets (p < 0,05). At 3 months, the CD68 infiltration was still the lowest in the leaflet with *p* > 0,05 but not statistically different than in the wall and in the muscle. The CD68 infiltration significantly decreased at 3 months in the muscle (*p* = 0,000) (Fig. 5c).

##### Calcifications

Von Kossa staining was positive for one sinus at 3 months.

##### Remodeling

**Fig. 6:**
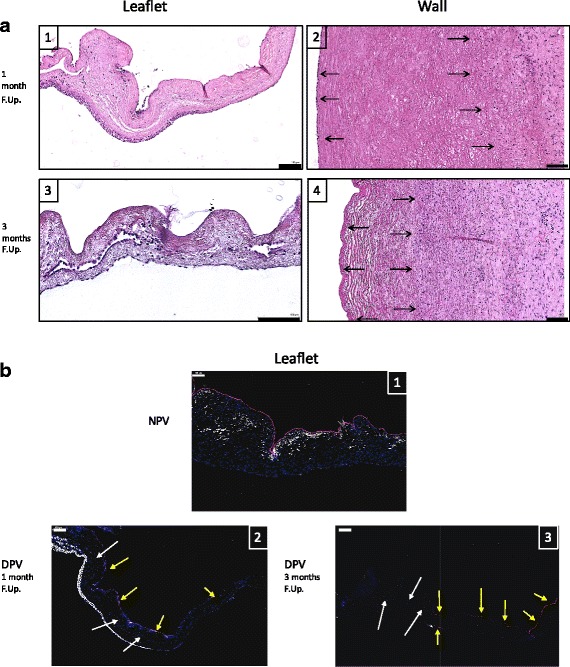
In vivo DPV Remodeling. **a**: Representative histological findings of Hematoxylin and eosin staining of DPV Leaflet (1,3) and Wall (2,4) of at one month (1;2) and three months (3;4) of follow-up illustrating the recellularization process. The black arrows indicate cell nuclei. Scale bar: 100 μm. **b**: Representative histological findings of CD31 (red) and ASMA (white) IHF stainings counterstained with DAPI (blue) of NPV leaflet (1), DPV leaflet at one month (2) and DPV leaflet at three months (3). Scale bar: 100 μm. The yellow arrows indicate CD31 + cells and white arrows DAPI + cells

HE, AMSA and CD31 staining showed progressive DPV recellularization occurred at one and 3 months (Fig. 6). The interstitial recellularization increased with time and cell colonization, was deeper in the pulmonary trunk at 3 months than at 1 month but was still partial (Fig. 6a). In a similar way, endothelialisation (endothelial cell monolayer) of DPV was still partial at 1 month and 3 months (Fig. 6b).

## Discussion

The aim of the present study was firstly to assess the efficacy of a NaOH-based process to decellularize and maintain biochemical and mechanical properties of xenogeneic valve in comparison to a standard detergent treatment. This treatment, a combination of 0.5%SDC /0.5%SDS is largely recommended for xenogeneic tissues decellularization as for human valves decellularization with good midterm clinical results in pediatric and adult populations [14, 23, 27–29]. Secondly a preliminary study investigated in a growing model biocompatibility and remodeling/cell recolonization of NaOH-based processed valve.

The main decellularization agent of our treatment is NaOH. The duration of exposure of the tissue to this agent and the combination with other chemicals offer a supplementary property to inactivate conventional (bacteria and virus) and non-conventional (prion) pathogen agents that a conventional detergent process does not offer, improving grafts security [22]. To our knowledge, this method for valve decellularization was not yet reported. The NaOH, while known as a decellularization agent, is not commonly used for biological tissue decellularization because it is at risk to denature the tissue. Indeed, decellularization treatments have to respect a perfect balance between decellularization and maintenance of physical properties for optimal in vivo functionality [30, 31]. Our in vitro and in vivo results showed the treatment did not denature the valves and can also ensure this balance for very thin structures such as leaflets.

Moreover, our treatment achieved better results in terms of decellularization and antigen removal than the detergent-based process. The presence of alpha-gal epitope remaining should lead to rapid deterioration of the last in a clinical scenario as it was observed for Synergraft valves [12, 32].

The NaOH-based process led to lower biochemical modifications of the valves in comparison to detergent treatment while mechanical properties of detergent-based processed valves were maintained.

It was shown that SDS might destabilize the triple helical domain of collagen and lead to tissue deterioration [33–35]. We noted the use of SDS lead to extracellular matrix swelling due to destruction of extracellular glycosaminoglycans as in our study while we did not observe direct consequences on mechanical tests not altered in comparison to native valves.

Additional studies also showed that the cytotoxicity of SDS can have an influence on the ingrowth of host valvular endothelial and interstitial cells [6, 36].

On the contrary, the NaOH-based process showed previously good clinical results regarding biocompatibility and cytotoxicity for other allogeneic biological tissues in different implantation sites with good host cell incorporation and remodeling [18, 19, 37, 38].

Our in vivo study confirmed this aspect. The in vivo results showed very low inflammation while the leaflets were thin and translucent at 3 months. Moreover, a recellularization, which is also of major concern for decellularized scaffolds integration and function, occurred and was progressive but still partial at 3 months. This phenomenon was also observed by others and a longer follow-up will investigate if complete recellularization can be achieved [39].

Another crucial point for cardiovascular prosthesis assessment is calcification occurrence. In this aspect, of the three implanted valves, in this growing model we observed one calcification focus in a sinus at 3 months. One explanation is although histologic examinations showed adequate decellularization and antigen removal, DNA quantification revealed only 92% DNA reduction in comparison to native in this region. This is less than the recommended threshold of 95% DNA reduction as nulceic acid can act as nucleation sites for calcifications [1, 16]. Unfortunately, a link is difficult to establish due to the small sample which constitutes a limit in our study.

Additionally, acetone and ethanol are part of the process. As detergents, these chemicals are recognized as antimineralization agents in heart valve substitutes by removal of phospholipids and cholesterol [1, 40–42].

Alcohols also aid in tissue decellularization by dehydrating and lysing cells. However, alcohol and acetone as tissue fixatives can damage ECM ultrastructure. In comparison to detergent treatments, acetone and alcohol crosslink ECM can produce stiffer scaffolds with mechanical properties further removed from those of native tissue [16]. We did not observe this phenomenon and conclude the proposed dilutions and duration of tissue exposure are not deleterious.

Last, the preservation method that we used is simple, cost effective, and the valve can be easily stored and banked for a long time as musculo-skeletal tissues in a tissue bank [18].

The results of this preliminary study are encouraging to consider this NaOH-based process for xenogeneic valve decellularization. In the clinical setup, xenogeneic source is advantageous regarding availability. But xenogeneic tissue transplantation to humans imposes high caution in view of controversial results with xenogeneic decellularized gafts [12]. We investigated only alpaha gal epitope removal. Others xeno non alpha-gal antigens exist and especially a recently highlighted, highly immunogenic xenoantigen, N-glycolylneuraminic acid antigen. This should be also investigated. The new genetically modified pigs for these major xenoantigens (alphagalactosyltransferase KO N-glycolylneuraminic acid KO pigs) offer new possibilities in this direction [43]. On the other hand, it would be impossible and inappropriate to check the disappearance of all xeno antigens [44] and, as suggested by G Gerosa, a step back to the preclinical evaluation in human-like models (non-human primates) is mandatory to assess effective biocompatibility [17].

Moreover, larger samples with a longer follow-up and echocardiographic data are prerequisite before considering clinical translation.

## Conclusions

The NaOH-based process does not alter biomechanical valve properties and can be used for xenogeneic heart valve decellularization. It ensures better decellularization and antigen removal than detregent-based process. In a preliminary in vivo study, the NaOH-based processed valve showed recellularization, low inflammation, and absence of structural deterioration. Regarding additional property of graft securing, this treatment should be considered for the new generation valves.

## Abbreviations

alpha-gal: Alphagalactosyltransferase antigen
ASMA: Alpha smooth muscle cells actin
BSA: Bovine serum albumin
DAPI: 40.6-diamidino-2-phenylindole
DDPV: Decellularized porcine pulmonary heart valves with detergent-based process
DNA: Desoxyribonucleic acid
DPV: Decellularized porcine pulmonary heart valves with NaOH-based process
HE: Hematoxylin and eosin
MHC-1: Major histocompatibility complex class 1
NPV: Native porcine pulmonary heart valves
PBS: Phosphate buffered saline
SD: Standard deviation
SDC: Sodium deoxycholate
SDS: Sodium dodecyl sulfate
SLA-1: Swine leucocyte antigen: MHC class 1 region of pigs
